# Application of CRISPR/Cas9 in Understanding Avian Viruses and Developing Poultry Vaccines

**DOI:** 10.3389/fcimb.2020.581504

**Published:** 2020-11-24

**Authors:** Julianne Vilela, Mohammed A. Rohaim, Muhammad Munir

**Affiliations:** Division of Biomedical and Life Sciences, The Lancaster University, Lancaster, United Kingdom

**Keywords:** CRISPR/Cas9, avian viruses, recombinant vaccines, genome editing, poultry diseases

## Abstract

Clustered regularly interspaced short palindromic repeats associated protein nuclease 9 (CRISPR-Cas9) technology offers novel approaches to precisely, cost-effectively, and user-friendly edit genomes for a wide array of applications and across multiple disciplines. This methodology can be leveraged to underpin host-virus interactions, elucidate viral gene functions, and to develop recombinant vaccines. The successful utilization of CRISPR/Cas9 in editing viral genomes has paved the way of developing novel and multiplex viral vectored poultry vaccines. Furthermore, CRISPR/Cas9 can be exploited to rectify major limitations of conventional approaches including reversion to virulent form, recombination with field viruses and transgene, and genome instability. This review provides comprehensive analysis of the potential of CRISPR/Cas9 genome editing technique in understanding avian virus-host interactions and developing novel poultry vaccines. Finally, we discuss the simplest and practical aspects of genome editing approaches in generating multivalent recombinant poultry vaccines that conform simultaneous protection against major avian diseases.

## Introduction

Poultry offers a major food source worldwide and continues to expand to satisfy the rising demands of the global population (Speedy, 2003). However, industrialization of poultry production and high expectation set the bases of expanded disease burden on poultry birds (Collett et al., 2020; Imran et al., 2020). Of the various infectious diseases in poultry, viral infections are the most impactful and are perhaps the most important stresses on the poultry productivity (Brun, 2016; Habiba et al., 2020). Chicken is a useful model for different fields of research, particularly for avian transgenesis and genome editing (Chojnacka-Puchta and Sawicka, 2020). The generation of genetically modified chickens is now much more achievable than ever, and precision genome targeting has recently been achieved (Macdonald et al., 2012; Tyack et al., 2013). Additionally, there is a growing interest in producing genetically engineered chickens that are resistant to diseases and are capable of high production (Sid and Schusser, 2018). Nevertheless, vaccines remained the primary means of disease prevention through immunization regimes in the poultry sectors.

Advances in pathogens biology and recombinant DNA technology are setting foundations in providing novel approaches to overcome the problems encountered with conventional vaccines design (Khan et al., 2016; Nadeem et al., 2020). Vaccine production has continued to be influenced by conventional vaccine preparation methods such as the use of existing well-characterized vectors, expression of discrete viral proteins and/or component(s), and production of virus-like particles (VLPs). However, novel approaches such as reverse genetic systems and genome editing technologies (i.e., CRISPR/Cas9) are currently being utilized to overcome the challenges in establishing an immunogenic platform that is safe and able to induce long-term immunity (Lauring et al., 2010; Lino et al., 2018). The development of CRISPR/Cas9 fuelled the biological and biomedical studies in different cell types and living organisms through means of a range of efficient and flexible genetic modifications due to their scalability, affordability and versatility in engineering (Cong et al., 2013). This innovative genome editing tool have created a paradigm shift in the life sciences and was selected by Science magazine as the “breakthrough of the Year” 2015 (Travis, 2015). The CRISPR/Cas systems are categorized into six major groups (Type I–VI) and at least 25 subtypes based on the Cas gene signature and the specific targeting mechanism (Koonin et al., 2017). Out of two classes (Wright et al., 2016), the class 1 system consists of diversified Cas systems—type I, type III, several archaic Cas but less popular in bacteria, as well as the uncommon type IV which includes primitive CRISPR-Cas without the adaptation module. On the other hand, the class 2 system is simple in organization compared to class 1, with a single, large, multidomain, and multifunctional protein in the effector module and contains the common type II (Cas9, the key tool in genome editing). The much rare types, V and VI, each with a special large-effector protein architecture (Koonin et al., 2017) are relatively less studied. Groups I-III include a well-studied systems including Cas3, Cas9, and Cas10 while group IV-VI are more recently characterized including Csf1 (Makarova and Koonin, 2015), Cpf1, C2c1, or C2c3 (Shmakov et al., 2015; Zetsche et al., 2015), and C2c2 (Shmakov et al., 2015). Group I, III, and IV Cas systems are classified within class I system and utilize a collection of Cas proteins. Group II, V, and VI Cas systems belong to class II wherein a single Cas9 protein performs the searching, binding, and cleaving of the target DNA sequence (Makarova et al., 2015; Shmakov et al., 2015). While Cas proteins carry complex structure and functional implications, these offer fertile opportunities in genome editing, biotechnology, and myriad of other disciplines in biomedicine.

This review highlights the recent advancements in the application of CRISPR/Cas9 system in the field of avian virology, and the most recent knowledge in the application of the technology in developing advanced, multiplexed, and novel vaccines against major viral poultry diseases.

## CRISPR/Cas9 Genome Editing System

Before the discovery of the RNA-guided endonuclease CRISPR/Cas9, genome editing in animals was carried out by mutagenesis (using chemicals and UV), nuclease-mediated [using zinc-finger nucleases (ZFNs), transcription activator-like effector nucleases (TALENs)], and recombinase-mediated (using Cre recombinase) (Rocha-Martins et al., 2015). In 2007, CRISPR was first described and defined as an unintended method mechanism of protection used by bacteria to suppress viruses (Travis, 2015). In 2012, the mode of action of the Cas9 protein was first demonstrated *in vitro*, showing its ability to cleave different DNA sequences through small synthetic RNA (Gasiunas et al., 2012). Subsequently, *in vivo* experiments started in 2013 wherein multiple laboratories demonstrated rapidly the ability of CRISPR-mediated genome editing in mammalian cells (Cong et al., 2013; Jinek et al., 2013; Mali et al., 2013b).

Through its simplicity and effectivity, CRISPR/Cas9 technology has paved the way in revolutionising the world of genome editing and modifications that were difficult or not feasible using previous technologies (Mali et al., 2013a; Doudna and Charpentier, 2014; Hsu et al., 2014; Sander and Joung, 2014). CRISPR/Ca9 system provides heritable adaptive immunity to prokaryotes to counter invading foreign genetic elements (i.e., viruses and plasmids) (Barrangou and Marraffini, 2014). The naturally occurring CRISPR system is composed of the following components: (1) Cas operon or cas genes, (2) a AT-rich leader sequence, and (3) arrays of repeats separated by unique spacer sequences (Bayat et al., 2018). In the CRISPR system, these repeats are interspaced by non-repetitive unique spacer sequences, which are primarily DNA fragments acquired from previous invading virus or bacteriophage (Bayat et al., 2018). The organism's resistance to foreign invading elements is conferred by processing of the transcripts from the spacer sequences to generate a 20 bases long small RNAs or CRISPR RNA (crRNA) which facilitates the binding and cutting of the Cas9 endonuclease enzyme at a precise location within the double-stranded DNA of the foreign virus or bacteriophage. This process is followed by deletion or addition of the DNA fragments to the CRISPR spacer array (Jinek et al., 2012). In order to initiate a precise double-stranded break in the invading foreign DNA, the endonuclease enzyme Cas9 forms an RNA duplex complex; crRNA: tracrRNA. These crRNA: tracrRNA complex can be synthetized and modified as what is now known as single guide RNA (sgRNA) that retain its fundamental properties: a nucleotide sequence that recognizes the DNA target site (crRNA) and a duplex RNA and able to binds to Cas9 (tracrRNA) (Doudna and Charpentier, 2014). Using this modified system, Cas9 can target a specific region by the sgRNA, wherein Cas9 induces a double-stranded break (DSB) (Gasiunas et al., 2012; Jinek et al., 2012), which can be repaired through either the error-prone non-homologous end joining (NHEJ) or repair-template guided homology-directed repair (HDR) (Rudin et al., 1989; Rouet et al., 1994). The development of simplified components for the CRISPR/Cas9 system allows a precise, efficient and user-friendly technology for editing and modifying genomes for a wide array of applications across multiple discipline including virology.

### Applications of CRISR/Cas9 in Virology

#### Studying the Virus-Host Interactions

Studying the host factors plays an important role in understanding the different aspects of cellular biology and virus-host interactions, which can lead to the development of new antiviral treatments (Puschnik et al., 2017). Likewise, it is important to consider the mechanisms by which viruses cause disease to direct optimum and appropriate design of tailored therapies and vaccines. So, understanding the mechanisms by which viruses infect, replicate, or spread are essential to guide in the development of precise target to prevent infection (Puschnik et al., 2017). The CRISPR/Cas9 has been employed in order to classify specific genes and proteins contributing to pathogenesis of various pathogens (Doerflinger et al., 2017). For efficient viruses' replication, they manipulate the host cellular receptors for entry and hijack cellular functions, construct virions of progeny and propagate. Recently, CRISPR/Cas whole genome screening have been utilized to identify the host factors essential for viral replication of some RNA viruses such as Dengue virus, Zika virus, West Nile, and hepatitis C (Puschnik et al., 2017). For example, utilizing CRISPR sgRNA library in the genome-wide screening of West Nile virus (WNV) revealed that the functional role of seven genes (*EMC2, EMC3, SEL1L, DERL2, UBE2G2, UBE2J1, and HRD1*) when being inactivated can induce protection against WNV-induced cell death (Ma et al., 2015).

#### Studying the Viral Gene Function

Investigating the basis for strain-specific differences provide an important information in the elucidation of viral component function and mechanism of viral process (Finnen and Banfield, 2018). Using CRISPR/Cas9, Luo et al. have developed a new framework for the modification of viral miRNAs encoded in the oncogenic alpha herpes virus (Marek's disease virus serotype 1, MDV-1) (Luo et al., 2020). Additionally, CRISPR/Cas9 have been applied to modify a series of viral miRNAs in the oncogenic MDV-1 genome in order to develop a simpler and more efficient, fast, and fairly non-disruptive platform to mutagenize minor genes such as miRNAs encoded by the herpesviruses and to study the functions of small non-coding RNAs. Briefly, two sgRNAs were used to facilitate the targeted manipulation of the viral miRNAs that allowed better understanding the MDV biology through the regulatory role of small RNAs and proven that exosomes can transfer viral miRNAs from MSB-1 cells to primary CEF cells in order to perform regulatory functions (Luo et al., 2020). Likewise, previous studies have developed a series of MDV transformed cells, expressing Cas9 in stable form (MSB-1-Cas9 and HP8-Cas9), and has demonstrated a gene editing strategy involving the transfection of synthesized gRNAs using a double part guide RNA system that efficiently deletes miR-M4 from the integral viral genomes (Zhang et al., 2019a,b). By employing CRISPR/Cas9, it was able to overcome some of the challenges in manipulating large DNA viral genome to study the viral function. Due to its simplicity, CRISPR/Cas9 platform can also be used in the characterization of other avian viral genome and its gene components which will not only be beneficial in avian viral genome biology but also in the development of viral vector vaccine, ensuring that the transgene is only integrated in the region that is not detrimental to the virus.

#### Antiviral Therapy

Advances in CRISPR/Cas9 genome editing enabled a novel approach in the development of therapeutic antiviral treatment by precise targeting of specific viruses within the infected cells. Majority of the recent studies utilized CRISPR/Cas9 for antiviral treatment focus on targeting the viral genome region responsible for viral gene expression and replication (Roehm et al., 2016), viral structure, transformation, and latency (Wang and Quake, 2014; van Diemen et al., 2016; Yuen et al., 2018). Within the genome of Marek's disease virus (MDV) vaccine strain, an optimal single gRNA and Cas9 expression cassettes were inserted to demonstrate the application of CRISPR/Cas9 for antiviral therapy (Liu et al., 2020). Liu et al. have evaluated the efficacy of MDV as a CRISPR/Cas9-delivery system to directly target and impede the replication of avian leukosis virus J (ALV-J) genome *in vitro* and *in vivo* by using single guide RNA, which targets the ALV-J's long terminal repeats (LTR) (Liu et al., 2020). By screening several potential RNA (gRNA) target sites for the ALV-J genome, different optimized targets were identified that would effectively disrupt the viral genome and protect against ALV-J infections by generation of numerous patterns and eventually excised indel mutations within the ALV-J genome (Liu et al., 2020). The versatility and reliability of ALV-J excision by using CRISPR/Cas9 MDV-based delivery method lays the foundation for clinical trials as a strategy for treatment of chronic viral infections in chickens (Liu et al., 2020). The progress in antiviral therapeutics using CRISPR/Cas9 technology will contribute in developing cure against viral infections.

#### Vaccine Development

Infectious diseases triggered by existing and evolving viruses, continue to endanger our safety and economy (Josefsberg and Buckland, 2012). Vaccination is the most successful tool to avoid the emerging diseases, protecting millions of lives per year, but the ongoing lengthy and complicated path to create antiviral therapies and or is rather ineffective (Josefsberg and Buckland, 2012). More challengingly, by changing its genome architecture, viruses evolves constantly and rapidly to overcome the vaccine-induced immunity (Devaux, 2012). Traditional strategies for the development of vaccines, including attenuated and gene deletion vaccines require a multiple rounds of plaque purification or passages which are not able to meet the urgent requirements for new vaccines especially during pandemics (Liang et al., 2016). Production of inactivated vaccines is more rapid and usually less expensive but needs a high dose administration (Liang et al., 2016). With the ongoing arms race with the rapidly evolving viruses, innovative developments are therefore urgently needed to improve the vaccine development to counter emerging and re-emerging viruses. The CRISPR/Cas9 offers a great level of solutions to challenge current traditional avian vaccine development strategies (Table 1).

**Table 1:** Comparison of CRISPR/Cas genome editing approach to conventional avian viral vaccine strategies.

**Parameter**	**Conventional approaches**	**CRISPR/Cas genome editing approach**	**References**
Virus plaque purification	Yes	No	Liang et al., 2016
Virus attenuation or inactivation	Yes	No	Liang et al., 2016
Gene-knockout/insertion	Complex	Simple	Tang et al., 2018, 2020; Atasoy et al., 2019; Chang et al., 2019
Diagnostic utility	Low	High	Tang et al., 2018, 2020; Atasoy et al., 2019; Chang et al., 2019
Simplicity	No	Yes	Tang et al., 2018, 2020; Atasoy et al., 2019; Chang et al., 2019
Feasibility	Difficult	Yes	Tang et al., 2018, 2020; Atasoy et al., 2019; Chang et al., 2019
Specificity	Low	High	Tang et al., 2018, 2020; Atasoy et al., 2019; Chang et al., 2019
Efficiency	Low	High	Tang et al., 2018, 2020; Atasoy et al., 2019; Chang et al., 2019
Cost	High	Low	Tang et al., 2018, 2020; Atasoy et al., 2019; Chang et al., 2019

The application of CRISPR/Cas9 provide a novel platform in the development of recombinant viral vaccines through improvements in vaccine design and experimental vaccination approaches across different species. CRISPR/Cas9 provide an alternative method to conventional approaches which is fast, efficient, and straightforward. Likewise, it has been applied for genetic modification in animal models (Ma et al., 2014), and genomic manipulation of several DNA viruses including herpes simplex virus type I, adenovirus, pseudorabies virus (Aujeszky's disease), vaccinia virus, Epstein–Barr virus, guinea pig cytomegalovirus, herpesvirus of turkey, and duck enteritis virus (Bi et al., 2014; Suenaga et al., 2014; Xu et al., 2015; Yuan et al., 2015; Bierle et al., 2016; Peng et al., 2016; Tang et al., 2016; Zou et al., 2017; Chang et al., 2018; Atasoy et al., 2019). The combination of CRISPR/Cas9 and Cre–Lox recombination techniques provides a powerful and versatile system to excise a pre-determined LoxP sites (34 base-pair DNA sequence), allowing the excision of specific genetic fragments and or selectable markers (Liang et al., 2016).

## Application of CRISPR/Cas9 GENE Editing in Avian Vaccines Development

Several approaches have been applied to edit viral vectors for the construction of recombinant vaccine candidates against poultry viruses (Baron et al., 2018). The current available methods for modification of viral vectored vaccines are inefficient, time-consuming, labor intensive especially for purification procedures (Zou et al., 2017). Thus, a more effective and reliable genome editing technology is urgently needed for the development of viral vectored vaccines. The CRISPR/Cas9 genome editing approach is the best choice as it did not only provide alternative ease and simple approach compared to traditional approaches, but it gives an opportunity to generate multivalent recombinant vaccines that confer simultaneous protection against major avian diseases.

To date, CRISPR/Cas9 has been successfully utilized in targeted mutagenesis of various viral vectors including herpesvirus of turkey (HVT) (Tang et al., 2018, 2020; Chang et al., 2019), infectious laryngotracheitis virus (ILTV) (Atasoy et al., 2019) and duck enteritis virus (DEV) (Zou et al., 2017; Chang et al., 2018). These recombinant viral vector genomes were edited to harbor and express specific antigens as potential viral vectored vaccines against multiples diseases such as NDV (Atasoy et al., 2019), AIV (Zou et al., 2017; Chang et al., 2019), MD (Tang et al., 2018, 2020), and ILTV (Tang et al., 2020). Likewise, the *in vitro* efficacy and stability of these recombinant vaccines had been evaluated through multiple cell passages compared to classical methods wherein transgene instability might result from multiple passages (Zou et al., 2017; Tang et al., 2018, 2020; Atasoy et al., 2019; Chang et al., 2019). To determine the stability of these constructs/cassettes during continuous cell passaging for the recombinant viruses for at least 15th passages and the inserts stability and expression were confirmed by western immunoblotting, immunofluorescence, and molecular detection (Tang et al., 2018, 2020; Atasoy et al., 2019; Chang et al., 2019). Therefore, these studies showed the potential of CRISPR/Cas9 as an effective, fast, and simple tool for the development of avian vaccines. The overview of CRISPR/Cas9 applications in avian viral vaccines construction is summarized in Table 2 and specifically discussed below for each virus.

**Table 2:** Overview of CRISPR/Cas9 applications in avian viral vaccines construction.

**Virus**	**Approach**	**Foreign viral antigen target**	**References**
Infectious laryngotracheitis virus (ILTV)	Gene knock-in and knockout	Fusion (F) gene of Newcastle disease virus (NDV)	Atasoy et al., 2019
Turkey's herpes viruses (HVT)	Gene knock-in	Glycoprotein D-glycoprotein I (gD-gI) of Infectious laryngotracheitis virus (ILTV), Hemagglutinin (HA) gene of avian influenza virus (AIV) and VP2 gene of Infectious bursal disease virus (IBDV)	Tang et al., 2020
		Hemagglutinin (HA) gene of avian influenza virus (AIV)	Esaki et al., 2013; Chang et al., 2019
		VP2 gene of Infectious bursal disease virus (IBDV)	Tang et al., 2018
Duck enteritis virus (DEV)	Gene knock-in	Hemagglutinin (HA) gene of AIV and the envelope glycoprotein (E) gene of duck tembusu virus (DTMUV)	Zou et al., 2017; Chang et al., 2018

### Newcastle Disease Virus (NDV)

Newcastle disease virus (NDV) is one of the most important avian viral pathogens causing major economic losses globally (Alexander, 2001; Zahid et al., 2020). NDV genetics and biology are extremely complex, with more than 20 currently proposed phylogenetically distinct genotypes based on the taxonomic classification (Dimitrov et al., 2019). Despite the continuous efforts to formulate an efficient ND vaccine, improvements are still required. In this pipeline, NHEJ-CRISPR/Cas9 system along with Cre/lox system offers a powerful approach to enhance and develop novel ND vaccines. We have demonstrated the safety and effectivity of infectious laryngotracheitis virus (ILTV) as a vaccine vector harboring the fusion (F) gene of velogenic NDV and showcase the versatility of NHEJ-CRISPR/Cas9 and Cre/Lox system (Atasoy et al., 2019). In these experiments, we deleted the thymidine kinase (*TK*) and unique short 4 (*US4*) genes from the ILTV genome and replaced with GFP reporter gene and F gene of NDV, respectively using NHEJ-CRISPR/Cas9 system to construct a bivalent vaccine candidate against two major avian respiratory viruses (ILTV and NDV). Thus, we concluded that the transgene insertion into the ILTV genome using CRISPR/Cas9 and subsequent excision of marker genes with Cre–Lox system is an effective approach to stably express genes without causing any detrimental effect for the viral vaccine vector replication (Atasoy et al., 2019).

### Avian Influenza Virus (AIV)

The widespread distribution of highly pathogenic avian influenza viruses (AIVs) causes substantial economic losses in the poultry industry while a persistent pandemic danger is posed by the occasional transmission of these viruses to humans (Peiris et al., 2007). The current vaccination strategies for AIVs serves as a preventive measure against virus infection in birds, rather than eradication (Collett et al., 2020; Irshad et al., 2020). However, despite the current efficacies of these vaccination strategies, birds are susceptible to influenza A viruses (Collett et al., 2020). CRISPR/Cas9 has been used in the development of virus-based vaccines against specific subtypes of avian influenza virus. Previous study demonstrated the use of CRISPR/Cas9 system as an effective and powerful tool for targeted genome engineering of Duck enteritis virus (DEV) to generate a vaccine candidate expressing the hemagglutinin (HA) protein of highly pathogenic avian influenza virus H5N1 (Zou et al., 2017). Moreover, this recombinant DEV vaccine also harbors two more genes [pre-membrane proteins (PrM) and envelope glycoprotein (E) genes] of the duck tembusu virus (DTMUV), generating a trivalent vaccine candidate against H5N1, DEV, and DTMUV infections in ducks.

The CRISPR-associated Cas9 achieves site-specific genome-engineering and can introduce a double-strand break (DSB) in a chromosomal location specified by the guide RNA (Cong et al., 2013; Jinek et al., 2013; Mali et al., 2013a). The DSB can be repaired either by non-homologous end joining (NHEJ) or homology-directed repair (HDR) pathway. The HDR pathway uses homologous donor DNA sequences from sister chromatids or foreign DNA for the production of exact insertions, simple substitutions between the DSB sites or two DSBs and other modifications whereas the NHEJ pathway creates correct deletions and insertions (Tang et al., 2019). The NHEJ pathway is a major type of DNA repair mechanism, divided into classical and alternative pathways that ligates the broken DNA together successfully (Mao et al., 2008). The NHEJ's low fidelity, which is likely to be error-prone, can result in base deletion or insertion (indel) after repair, resulting in a frameshift mutation (Bernheim et al., 2017). The NHEJ is the prevailing DSB-based repair route and accounts for most cell cycle DSB repairs (Arnoult et al., 2017).

Meanwhile, CRISPR/Cas9 error-free homology-directed repair (HDR) approach has been utilized to generate HVT-AIV bivalent vaccine and the selection of recombinant HVT-HA was done through the development of a precise, easy, and quick erythrocyte adsorption assay instead of using traditional approaches (Chang et al., 2019). This process yielded an approximate ~6% accuracy for H7N9 HA insertion indicating that NHEJ mediated the silencing of the majority of GFP negative HVT viruses (Chang et al., 2019). The CRISPR/Cas9-mediated DSBs can go through both NHEJ and HDR routes, but in most cases NHEJ is favored (Frit et al., 2014), thus the possibility of NHEJ-mediated silence can be justified. While HDR is a faithful pathway and can stimulate error-free repair mechanism, there is a need to enhance its efficiency.

### Marek's Disease Virus (MDV)

Marek's disease virus (MDV) is a lymphoproliferative disease of chicken primarily controlled by vaccination since 1969 (Okazaki et al., 1970). Turkey's herpes viruses (HVT), which belong to *Meleagrid herpesvirus* 1, have been widely used for more than 40 years as a vaccine against Marek disease (MD) (Okazaki et al., 1970). Recently, HVT is utilized as a viral vector to provide protective immunity against MD and other viral diseases through harboring and expressing foreign viral antigens of other viral diseases (Li et al., 2011; Vagnozzi et al., 2012; Esaki et al., 2013; Chang et al., 2019). CRISPR/Cas9 technology has revolutionized the HVT vaccine construction and the potential of HVT as a vector for multivalent vaccines. Tang et al., successfully generated a recombinant HVT with triple gene inserts leading to the development of a recombinant viral vaccine candidate that can potentially induce protection against three major avian viral diseases (ILTV, IBDV, AIV, MDV; Table 2; Tang et al., 2020). Similarly, Chang et al. used HDR-CRISPR/Cas9 to develop a recombinant bivalent HVT vaccine candidate expressing the hemagglutinin (HA) antigen of influenza A virus (Chang et al., 2019).

### Infectious Laryngotracheitis Virus (ILTV)

Infectious laryngotracheitis virus (ILTV) is a respiratory disease affecting the poultry industry and has a economic impact worldwide (Gowthaman et al., 2020). Infectious laryngotracheitis (ILT) disease caused by *Gallid herpsvirus type* 1 (GaHV-1) and can be transmitted through infected birds and fomites (Davison et al., 2009). Vaccination using attenuated and recombinant viral vectored vaccines is the primary control measure employed in the endemic regions (Collett et al., 2020). In an effort to develop more stable vaccine strains, CRISPR/Cas9 technology has been successfully applied to generate a recombinant viral vector expressing ILTV antigen (Table 2). Tang et al. have utilized CRISPR/Cas9 gene editing approach to knock-in the ILTV glycoprotein D-glycoprotein I (gD-gI) expression cassette into a specific region within the HVT genome (Tang et al., 2020). These findings showed that CRISPR/Cas9 gene editing approach was able to cut the HVT genome at a specific region and insert the ILTV gD-gI expression cassette through homology-independent NHEJ repair system. The authors also succeeded in inserting and expressing two more antigens—AIV H9 HA and IBDV VP2 genes into various locations of the recombinant HVT genome, developing a stable trivalent recombinant HVT vaccine.

## CRISPR/Cas9 Workflow for Vaccine Development

**Figure 1:**
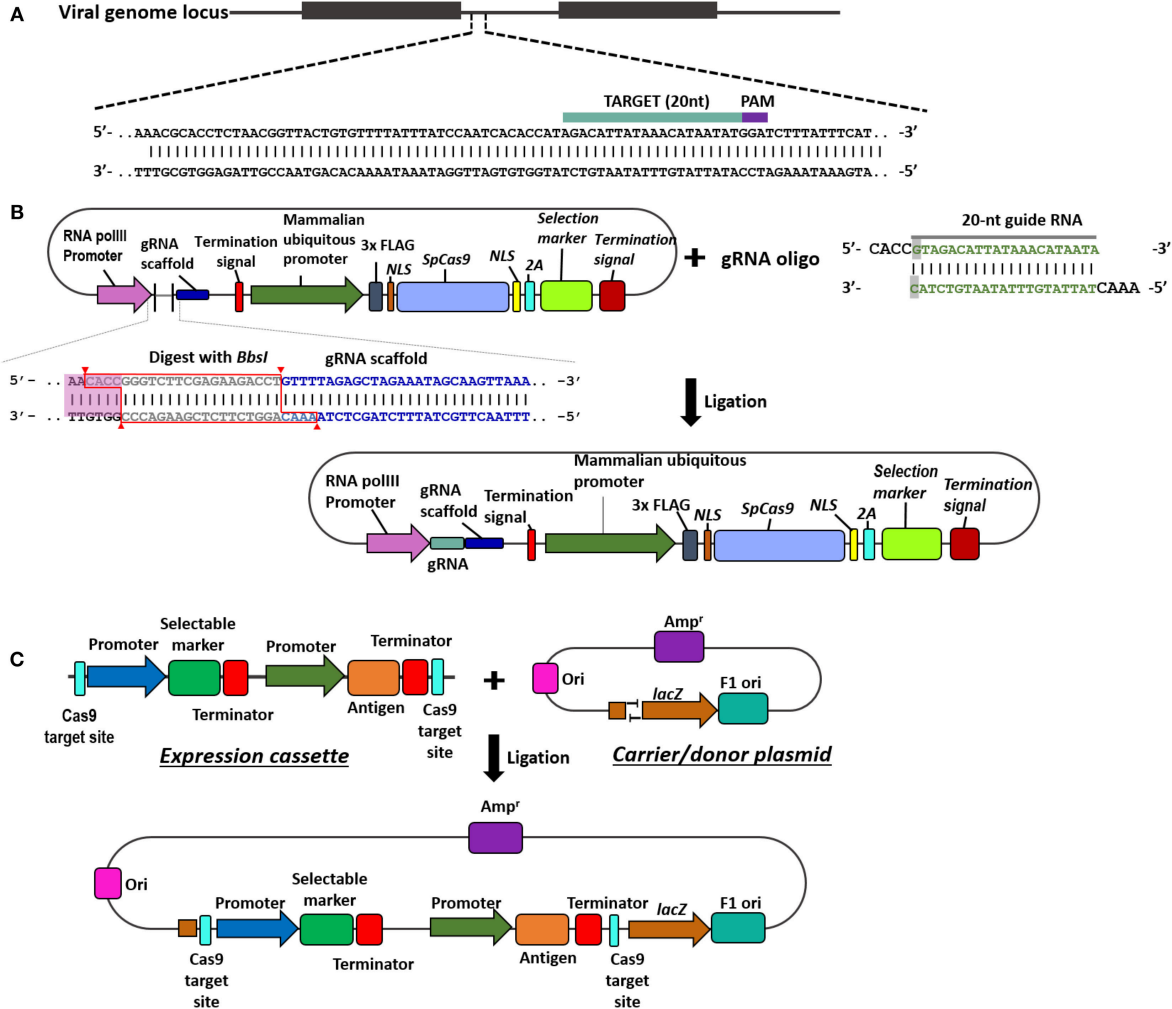
Overview of steps in the construction of CRISPR/Cas9 and donor plasmids. **(A)** The 20 nucleotides viral genome target (highlighted in teal) is followed by the PAM recognition site (highlighted in purple), 5′-NGG. **(B)** Schematic on the construction and assembly of the Cas9/gRNA expression plasmid. Digestion of the plasmid containing Cas9 and gRNA scaffold with BbsI allows the insertion of the annealed gRNA oligos (teal) through replacing the type II restriction site (outlined in red). **(C)** Schematic on the construction of the donor plasmid harbouring the antigen and selectable marker. The T overhangs on the carrier plasmid is complementary to the overhangs in the expression cassette to facilitate ligation.

Understanding the components of CRISPR/Cas9 technology in viral vector vaccine development is essential for the success to rescue recombinant vaccine. Figure 1 provides an overview of the *in silico* design of sgRNA, and experimental construction of CRISPR/gRNA and donor plasmids.

### Selection of Viral Vector and Target Insertion Site

Recombinant viral vectors are very powerful tool for developing vaccines and addressing experimental vaccination. Generally, virus can be used as a vector to express foreign genes. Poxvirus (both Orthopoxvirus and Parapoxvirus), Herpesvirus, Adenovirus, and Baculovirus are the most commonly used DNA viruses for the development of recombinant viral vectors (Brun, 2016), while Alphavirus, Bunyavirus, Coronavirus, Flavivirus, Paramyxovirus, Retroviruses, Rhabdovirus are some of the RNA viruses being utilized (Brun et al., 2008). The introduction of reverse genetics technologies enables us to rescue the infectious virus from a copy of its genome. The key benefit of DNA viruses rather than RNA viruses is due to the DNA genomic stability, multiple insertion sites, and availability of BAC clones for downstream laboratory applications. In general, the key elements in selecting viral vectors include: safe and effective expression of the antigen, low to moderate intrinsic immunogenicity to facilitate re-administration and/or booster vaccination, and scalability for production purposes (Haut et al., 2005). The use of CRISPR/Cas9 in generating viral vectors has been widely implemented in viral vectors such as adenovirus, herpesvirus, and poxvirus (Esaki et al., 2013; Zou et al., 2017; Tang et al., 2018, 2020; Atasoy et al., 2019; Chang et al., 2019). Among live replicating viral vectors, herpesvirus of turkeys (HVT) is the most commonly used viral vector for ovo- and subcutaneous hatchery administration because of its adaptability and safety and can provide a life-long immunity even in the presence of maternal antibodies. Recombinant HVT vaccines are either developed by traditional homologous recombination in virus-infected cells or by recombination approaches utilizing cosmid clones overlapping or cloned to full-length genomes as bacterial artificial chromosome (BAC) clones (Baigent et al., 2006; Li et al., 2011) which are also time consuming and ineffective. In addition to the protection against MDV, the majority of recombinant HVT vaccines currently in use in the poultry industry can individually and or dually express foreign gene inserts to induce protection against either one or two pathogens (Gergen et al., 2019). Although HVT vector-based vaccines are effective and increasingly used by industry, interference between the HVT vector backbones severely limits the simultaneous usage for sets of HVT vector vaccines expressing antigens against multiple diseases (Dunn et al., 2018). Tang et al. have successfully generated trivalent HVT based vaccine by insertion of glycoprotein D-glycoprotein I of ILTV, HA of H9N2 avian influenza virus and glycoprotein VP2 of IBDV, generating recombinant HVT-VP2-gDgI-HA with triple inserts (Table 2; Tang et al., 2020).

#### Choosing Target Insertion Site

Advances in next generation sequencing platforms have enabled the sequencing of species entire genome and viruses's genome. The National Center for Biotechnology Information (NCBI) dedicated a web-based portal for the collection of virus sequences and datasets (Hatcher et al., 2017). The primary consideration to choose a target region for CRISPR/Cas9 knock-in is maintaining the ability of virus vector replication (Yajima et al., 2018), thus potential transgene insertion sites must be well-characterized and verified to have a minimal effect on viral replication.

#### Choosing Viral Antigen

It is important to understand the mechanism of viral infection and pathogenicity. Most of the points related to choose viral antigen have already been described, although it is necessary to consider whether a single antigen can produce sufficient protective immunity or whether multiple antigens are required (Chambers et al., 2016).

#### Selection of Host Cell Lines

Cultured cell lines are excellent hosts for the propagation of many types of viruses. Since viruses are obligate intracellular parasites, they depend on hosts for survival. For the use of the CRISPR technology, the viral vector needs to be grown in a living host to support its replication in order to target its genome. The main cell lines used for the production of viral vaccines remains to be the established animal cells which includes Vero, MDCK or chicken embryo fibroblasts (CEFs) (Genzel, 2015). On the other hand, for the generation of various avian viruses such as AIV, IBV, MDV, avian metapneumovirus (AMPV), and infectious laryngotracheitis virus (ILTV), immortalized avian cell line Leghorn male hepatoma (LMH) has been commonly used (Schnitzlein et al., 1994).

#### Construction of sgRNAs, Donor Plasmid, and Cas9/gRNA Expression Plasmid

Using CRISPR/Cas9 technology in the development of recombinant viral vectors requires understanding the following: (1) *in silico* design for the gRNA to target the viral genome; (2) construction and assembly of the Cas9 and sgRNA expression vector system and (3) construction of the antigen and selectable markers expression cassette and assembly to the donor plasmid. The Cas9 endonuclease precision is dependent on single guide RNA (sRNA) complementary to the target sequence. In designing the sgRNA, primary considerations are the PAM region specific for S. *pyogenes* Cas9 recognition which is 5′-NGG and the on/off-target activity. Various web-based and local programs have been developed to identify possible PAM region and target sequences, as well as provide a predictive value that ranks the gRNAs based on on-target and off-target activities. Table 3 provides a list of existing free online tools and open-sources local software suitable for viral genome editing. It is also important to validate the off-target activity of sgRNA in the host cell genome (i.e., Chicken).

**Table 3:** List of existing free online tools and open-source local software for sgRNA design.

**Name**	**Graphical user interface**	**Searches whole genome for targets**	**Input**	**Output**	**Predicts gRNA activity**	**Ranked list**	**References**
CHOPCHOP	Yes	Yes	Gene ID, chromosome position; also allows gene input	Candidate guide sequences and off-target loci	No	Yes	Montague et al., 2014
CHOPCHOP v2	Yes	Yes (over 200)	Gene ID, chromosome position; also allows gene input	Candidate guide sequences and off-target loci	Yes	Yes	Labun et al., 2016
Cas-OFFFinder	Yes	Yes (over 400)	Guide sequence	Off-target loci for guide sequences	No	No	Bae et al., 2014
CasOT	No (Perl script)	User input	Guide sequence	Off-target loci and additional guide sequences	No	No	Xiao et al., 2014
Benchling	Yes	Yes	DNA sequence or gene name	Candidate guide sequences and off-target loci	No	Yes	Benchling, 2020
CRISPOR	Yes	Yes (over 200 genomes)	DNA sequence	Candidate guide sequences and off-target loci	Yes	Yes	Haeussler et al., 2016

Cas9 enzyme and gRNA need to be expressed in the cells for the efficient CRISPR/Cas9 technology. Various plasmid expression systems are available to use CRISPR in eukaryotic cells. To select the plasmid expression system, some precautions should be considered such as Cas9 enzyme and gRNA expression promoter, plasmid backbone components including selectable markers (antibiotic or flourophore) and appropriate plasmid delivery method. Addgene (2020) (https://www.addgene.org/crispr/) provides a wide array of CRISPR plasmids and resources.

Meanwhile, it is essential to have a donor plasmid that carries the antigen gene and selectable marker cassettes for efficient CRISPR/Cas9 technology to develop recombinant viral vector vaccine. The donor plasmid will provide the necessary DNA fragment to facilitate a homology-independent targeted integration in the genome target site (Pickar-Oliver and Gersbach, 2019). Donor plasmids are constructed following traditional cloning techniques, specific fragments such as promoters, terminators, selectable markers, and antigens are either PCR-amplified or synthetically generated. These fragments are then incorporated into a plasmid backbone through digestion-ligation reaction.

### Generation of Recombinant Viral Vector

**Figure 2:**
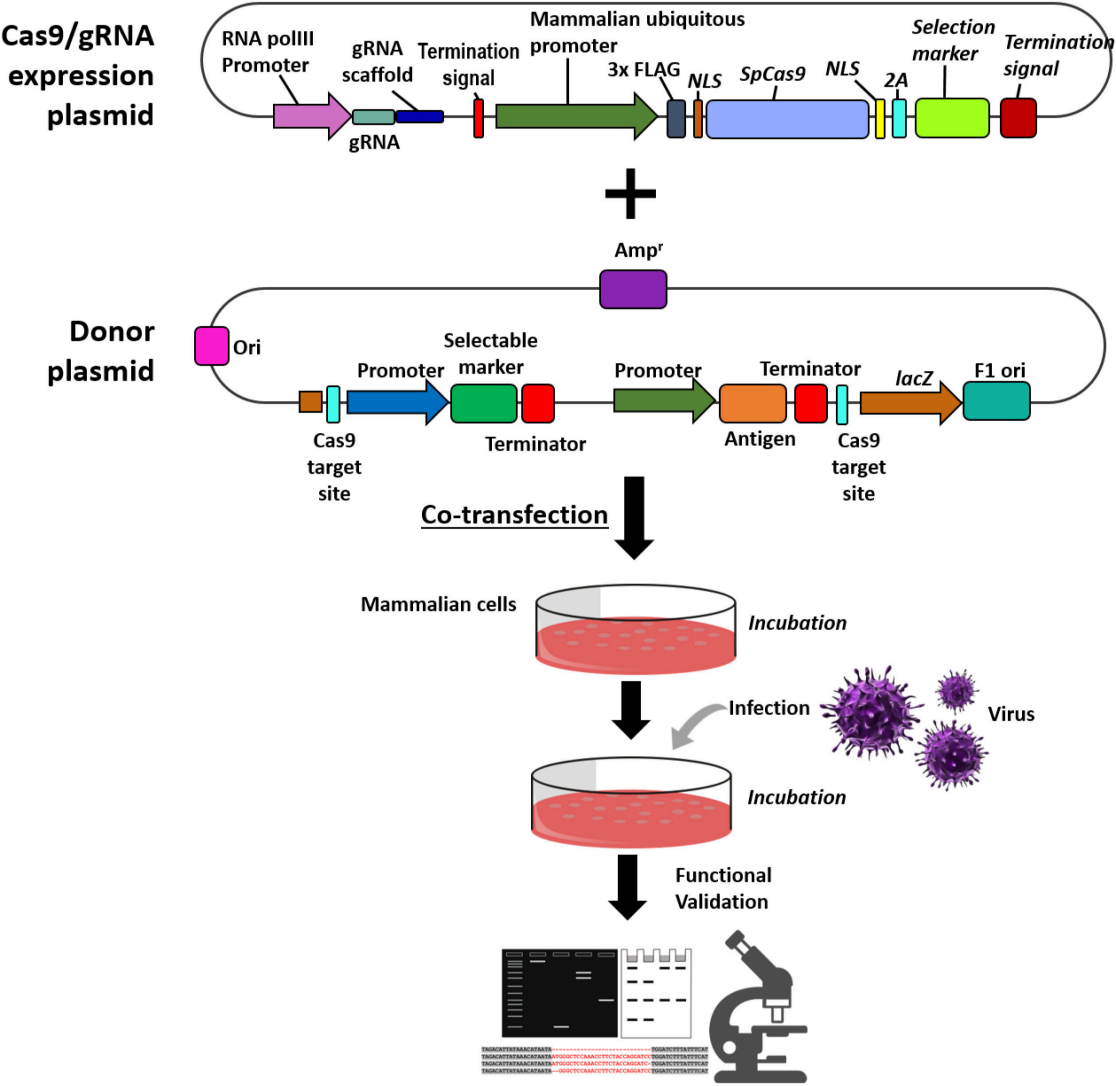
Schematic on the generation of recombinant viral vector and functional validation. Steps for the co-transfection of the Cas9/gRNA expression cassette and donor plasmid to the mammalian cells, followed by infection of the virus of interest. Functional validation and characterization of the recombinant viral vector through western blot analysis, PCR amplification and microscopy.

In the CRISPR workflow, the delivery of the CRISPR components is an essential step. Figure 2 is a schematic on the generation of recombinant viral vector and functional characterization. The delivery of these components into eukaryotic cells is facilitated by transfection, which can be broadly categorized into physical, chemical, and viral-mediated. These methods have different advantages and disadvantages in terms of efficiency, performance, equipment, skills, and cost. Physical delivery method utilizes energy supplied by various forces (i.e., electric, thermal, and mechanical). These methods disturb the cell membrane and facilitate the entry of the CRISPR components to the cell. While physical transfection have its advantages it still remains too invasive for some applications (Fajrial et al., 2020). The viral-mediated delivery uses viral vectors to transfer the plasmid DNA to the host cell. While this delivery system is advantageous in many CRISPR experiments, for the purpose of vaccine development using viral vectors it might pose some challenges as the mechanism of antagonistic relationship between two or more viruses in the host cell is not yet fully explored. Lipofectamine is widely used chemical transfection methodology in CRISPR component delivery. The chemical vector serves as a delivery system encapsulating the plasmid DNA. Once the components are delivered, the cells are incubated at a range of 12 to 24 h (Tang et al., 2018; Atasoy et al., 2019). Optimization is essential to determine the appropriate incubation time, to ensure the proper binding of Cas9 to the gRNA to elicit an active Cas9 conformation (Raper et al., 2018). Once the components are transfected to the host cells and incubated, infection of the virus will follow. Incubation time in this step varies from 24 to 48 h, ample time is needed to facilitate the following steps in the CRISPR system (1) PAM recognition in the viral genome, (2) viral DNA unwinding and gRNA strand invasion, and (3) Cas9 conformational changes to initiate DNA cleavage (Sternberg et al., 2015). Cells are then incubated until the formation of fluorescence signals followed by cell passaging before they reach 70% confluency for downstream application, validation, and characterization.

### Characterization of Recombinant Viral Vector

Characterization of the recombinant viral vector can be conducted using PCR amplification, microscopy, and western immunoblotting. PCR-based amplification can validate the presence and direction of the insert. Early on during the construction of Cas9/gRNA and donor plasmids, oligo primers are designed to amplify specific regions of the plasmids and inserts for validation of the presence and correct size. Precise sequence information of the PCR-amplified DNA fragments can be identified through Sanger sequencing. Raw sequences are then analyzed using bioinformatics tools for multiple sequence alignment of clones against the reference sequence. The same method is used in the selection of recombinant viral vectors with the desired homologous gene edit.

To further characterize the recombinant viral vector after the CRISPR/Cas9 mediated integration of antigens, expression of functional antigen proteins can be observed using western blotting experiments. In this experiment cells are infected with the recombinant viral vector harboring the antigen. Characterization is achieved through the implementation of 3 major steps: (1) protein separation from the mixture through SDS-PAGE, (2) transfer of separated protein into a solid support such as nitrocellulose membrane, and (3) detection of the protein through binding to appropriate antibodies (Estep et al., 2016). To further characterize the expression of the antigen protein, immunofluorescence or cell imaging is employed. So, the expression of the antigen protein is carried out by the comparison of fluorescence between the wild-type and the recombinant viral vector.

## Perspective and Future Direction

In last few years, substantial improvements have been made in the application of genetic manipulation techniques to produce the desired vaccines. Recently evolved CRISPR/Cas9 technology is an important resource in a number of disciplines and has enabled extremely successful generation of gene-edited chickens. Recent studies have proven that CRISPR/Cas technology opens a new avenue in research for vaccine development through its efficiency, specificity, versatility, and simplicity. The development of multivalent vaccine vectors demonstrates a great potential for CRISPR/Cas system, which is an important area of continuous study to further develop novel avian viral vaccines. The best way for prevention and control of avian viruses in the industry can be achieved through vaccination. Through prevention as the most efficient means of preventing virus transmission, it increases the poultry welfare by minimizing tension associated with frequent needle injections, speeds up the vaccination procedures, decreases the production expense, and provides a promising opportunity to protect the poultry industry against multiple diseases with less doses from the vectored multivalent vaccine. Recombinant vectored vaccines are promising to be effective to vaccinate against multiple diseases. Traditional recombination methods or bacterial artificial chromosomes are used in the development of recombinant vaccines, but they can be time consuming. The CRISPR/Cas9 system has shown to be beneficial for gene modification and provides an alternative to recombinant vaccine construction. Two key methods are used for gene insertion; NHEJ (non-homologous end joining) error-prone and HDR (homology-directed reparation pathway) high fidelity. Due to its high degree of reliability, the main focus of most studies is on the HDR application for vaccine production. Our research investigated the fast generation of recombinant vaccines by both HDR and NHEJ based CRISPR/Cas9 systems. The CRISPR/Cas9 system is a gene-editing technology, which has been recently developed and has shown to be beneficial in gene modification and provides an alternative rapid development of recombinant vaccines based on HDR and NHEJ systems.

For multiple antigen insertion within the viral vector, the selection of an appropriate selectable marker is challenging especially with the use of fluorescent markers. The application of Cre/lox system to subsequently excise the fluorescent marker within the recombinant viral vector has been implemented and provided an answer to the challenge of selection of multiple antigen inserts and removal of the selectable marker for vaccine licensing. Development of a strategy for a single donor plasmid approach that carries multiple antigens will make a meaningful impact to the field. This is an important area of continuous study for the production of vaccines.

Further, while majority of the CRISPR/Cas9 studies focus on the improvement of its specificity and efficiency, it is also important to address the biosafety implications of the on and off target effects of the system. Biosafety studies should be carried out to study on and off-target effects and their possible unintended mutations on the host cells and the potential interaction with the virus. Outcomes of these studies will be beneficial in the proper utilization of CRISPR/Cas9 tool in the advancement of viral vector construction. Taken together, the CRISPR/Cas9 system provides a significant advancement in various areas in avian virology specially in vaccine development. Together with the improvement in modern technology, next-generation sequencing and artificial intelligence, CRISPR/Cas9 system will continue to improve and ultimately its limitations will be addressed and solved.

## Author Contributions

MM conceived the idea, has edited and reviewed the manuscript. JV and MR have written the manuscript. All authors approved the manuscript for submission.

## Conflict of Interest

The authors declare that the research was conducted in the absence of any commercial or financial relationships that could be construed as a potential conflict of interest.
